# Study of Extensional Rheology Behavior of Sodium Alginate/Polyethylene Oxide Solutions for Blow Spinning

**DOI:** 10.3390/ma18245491

**Published:** 2025-12-05

**Authors:** Biao Yang, Xue Wang, Cong Du

**Affiliations:** Shandong Key Laboratory of Renewable Membrane Materials, College of Materials Science and Engineering, Qingdao University, Qingdao 266071, China; yb19862511837@163.com (B.Y.); yawx128ve980@163.com (X.W.)

**Keywords:** extensional rheology, pinching dynamics, polysaccharide, solvent property, blow spinning

## Abstract

Blow spinning is a low-cost and versatile method that permits the large-scale production of fibrous membranes. However, polysaccharides that show numerous merits such as biocompatibility and biodegradability often have a low spinnability due to their high chain rigidity and low ability to form sufficient entanglements. Here, we report the fabrication of polysaccharide micro-fibrous membranes from sodium alginate/polyethylene oxide solutions formulated in solvent mixtures of water and ethanol. The shear and extensional rheological responses of the solutions are characterized, and parameters including specific shear viscosity, reptation time, extensional relaxation time, and maximum stretch ratio are correlated with the concentrations of polymer, polyethylene oxide, and ethanol. It is found that flexible polyethylene oxide and poorer solvent ethanol can synergistically delay the chain relaxation during stretch and increase the stretchability of the solutions. A processability map of the solutions for blow spinning is constructed, enabling the fabrication of fibrous membranes with a fiber diameter of ~1 μm, tensile strength of 4.89 MPa, elongation at break of 15.24%, and Young’s modulus of 45.43 MPa. This study presents a new strategy to fabricate sodium alginate-based membranes, which should provide insights into the design of other polysaccharide membranes with specific functions and applications.

## 1. Introduction

Facing the increasingly severe threat from plastic pollution, it is a promising strategy to substitute synthetic plastics with renewable, biocompatible, and biodegradable polysaccharides, including cellulose, chitosan, and sodium alginate (SA) [1,2,3,4,5]. Especially, electrospinning and blow spinning provide facile approaches to fabricate polysaccharide fibrous membranes that have demonstrated applications in adsorption [6,7,8,9], filtration [9,10,11,12], and heat insulation [13,14,15]. Flexible synthetic polymers such as polyethylene oxide, polyvinyl alcohol, and polyacrylonitrile that are often adopted to fabricate fibrous membranes for such applications are not biocompatible and biodegradable. Due to the semi-flexible nature of polysaccharide chains, the entanglement density of polysaccharide solutions is usually low, leading to poor stretchability and processability. The incorporation of flexible polymers with high molecular weight into polysaccharide solutions is a common strategy to enhance entanglement density and improve spinnability [16,17,18,19,20,21]. For example, Gao et al. introduced polyethylene oxide (PEO) into SA solutions to enhance the entanglements of SA chains, enabling the successful preparation of nanofiber membranes [22]. However, in these studies, the mass fraction of synthetic polymers is as high as 50 wt.%, compromising the biodegradability of polysaccharide membranes.

Adopting binary solvents to polysaccharide/synthetic polymer solutions is an effective way to decrease the concentration of synthetic polymers required for good spinnability [23,24,25,26]. The improved spinnability of polymer solutions with the addition of lower-polarity solvents is often associated with a faster evaporation rate and an enhanced entanglement density. For example, Dou et al. adopted ethanol as a co-solvent for SA/PEO solutions, achieving a successful preparation of nano-fibrous membranes with an exceptionally high SA content by electrospinning [6]. They proposed that the addition of ethanol not only reduced the conductivity of the solution but also promoted hydrophobic association and hydrogen bonding between polymer chains due to a poor solvent effect, enhancing chain entanglements. However, the type of solvent not only alters volatility, surface tension, viscosity, and dielectric constant of polymer solutions but also affects polymer–solvent interactions, polymer–polymer interactions, and polymer conformation. These influences on the spinnability of polymer solutions are often coupled and not well elucidated. Sharma’s group have shown that pinch dynamics and extensional rheology parameters of polymer solutions are correlated to structural parameters, including the flexibility and extensibility of polymer chains [27,28,29,30,31], providing a useful tool to disclose the fundamental mechanism of the spinnability regulation through solvent properties for polymer solutions.

Here, we adopt blow spinning to fabricate polysaccharide fibrous membranes from sodium alginate/polyethylene oxide solutions with the addition of ethanol (SA/PEO/EtOH solutions). The shear and extensional rheology responses of SA/PEO/EtOH solutions are systematically characterized using a torsional rheometer and a capillary breakup extensional rheometer (CaBER), respectively. It is found that the specific shear viscosity, shear relaxation time, and extensional relaxation time of the solutions all rise with polymer concentration and PEO concentration, indicating an increase in entanglement density. With the increased ethanol concentration, the specific shear viscosity and extensional relaxation time increase, indicating enhanced polymer–polymer interactions, whereas the specific terminal Trouton ratio and apparent maximum stretch ratio both show a maximum at ethanol concentration of 5 wt.%, revealing the increase in ethanol concentration initially promotes but subsequently hinders the stretchability of polymer solutions. By optimizing the solution compositions and blow spinning parameters, micro-fibrous membranes with good morphology and mechanical performance are formed. This work highlights the low-cost and facile processing of polysaccharide membranes by regulating solvent properties, which should open up new opportunities for the applications of polysaccharide materials in various fields.

## 2. Experimental Section

### 2.1. Materials

Sodium alginate (SA, *M*_w_: 4.1 × 10^5^ Da, G/M = 2) was provided by Qingdao Hyzlin Biology Development Co., Ltd. (Qingdao, China). Polyethylene oxide (PEO, *M*_w_: 1.8 × 10^6^ Da) was purchased from Shanghai Aladdin Biochemical Technology Co., Ltd. (Shanghai, China). Ethanol (EtOH, ≥99.7%) was received from Wuxi Feihong Chemical Industry Co., Ltd. (Wuxi, China). Millipore deionized (DI) water was used in all experiments. All chemicals were used as received without further purification.

### 2.2. Preparation of SA/PEO/EtOH Solutions

Prescribed amounts of SA and PEO powders were dissolved in DI water with mechanical stirring for 12 h to form homogenous solutions. The compositions of SA-*c*_p_-*f*_PEO_-*f*_EtOH_ solutions are shown in Table 1. A very slow stirring rate of 50 r/min was adopted to avoid the chain scission. Prescribed amounts of ethanol were then slowly dropped into SA/PEO solutions under slow mechanical stirring for another 12 h. The resultant homogenous solutions are coded as SA-*c*_p_-*f*_PEO_-*f*_EtOH_, where *c*_p_, *f*_PEO_, and *f*_EtOH_ are the total polymer concentration in wt.%, the mass fraction of PEO with respect to the mass of polymer concentration in wt.%, and the concentration of ethanol in wt.%, respectively.

### 2.3. Rheological Measurements

The steady shear viscosity of SA/PEO/EtOH solutions was measured using a DHR-3 rheometer (TA Instruments, New Castle, DE, USA) with a cone-and-plate fixture (diameter: 60 mm, cone angle: 1.998°, gap: 50 μm). Steady shear viscosity measurements were performed on the solutions in a shear rate range of 0.01–1000 s^−1^. The value of non-Newtonian index *n*_s_ can be obtained by fitting the data from shear-thinning regions using the following expression:

(1) $$\eta =K\;{\;\overset{\cdot }{\gamma }}^{n_{s}-1}$$

where *K* is the consistency index. Frequency sweeps were performed on the solutions with the angular frequency range of 0.1–500 rad s^−1^ and a strain amplitude of 1%. The extensional rheology behaviors of the solutions were obtained using a CaBER1 rheometer (HAKKE, Vreden, Germany). The solutions were loaded between two parallel plates with a diameter of 6 mm and an initial gap of 3 mm, and then pre-stretched to a final stretch gap of 18 mm with a striking time of 50 ms. After the upper plate stopped, the diameter of the liquid bridge midpoint was recorded over time. The apparent maximum stretch ratios were captured using a tensile test fixture constructed by a servo motor and a high-precision motorized translation stage. The solutions were loaded between two parallel plates with a diameter of 4 mm and an initial gap of 2 mm, and then stretched with a speed of 5 mm/s. The EC thinning dynamics can be quantitatively captured by the following equation [32,33]:

(2) $$\frac{D(t)}{D_{0}}\approx \left(\frac{G_{E}D_{0}}{4\sigma }\right)exp\left[-\frac{\left(t-t_{c}\right)}{3\lambda_{E}}\right],$$

where *D*_0_ is the initial liquid bridge diameter, *t*_c_ is the onset time for EC regions, *G*_E_ represents the extensional modulus, and *λ*_E_ is the extensional relaxation time that characterizes the time required for chain relaxation after stretching. The combined behaviors of the EC and TVEC responses in sequence are typically fitted using the equation proposed by Anna and McKinley [34]:

(3) $$\frac{R(t)}{R_{0}}=A\exp\left(-B\left(t-t_{c}\right)\right)-C\left(t-t_{c}\right)+D,$$

where the parameter *B* (*B* = 1/3*λ*_E_) represents the characteristic scale of the extensional relaxation time, and the parameter *C* = *σ*/(*D*_0_$\eta_{E}^{\infty }$) is related to the surface tension *σ* and steady terminal extensional viscosity $\eta_{E}^{\infty }$. To further clarify the effects of the component of SA/PEO/EtOH solutions on extensional rheology behaviors, the apparent extensional viscosity *η*_E_ is calculated by the following equation:

(4) $$\eta_{E}=\frac{2\sigma /D\left(t\right)}{d\varepsilon (t)/dt\;},$$

where *ε* represents the Hencky strain with *ε*(t) = 2ln(*D*_0_/*D*(*t*)), and *σ* is the surface tension. The capillary thinning and stretch-to-rupture procedures were recorded by an M230 high-speed camera (Revealer, Hefei, China) with a resolution of 1920 × 1080 pixels and frame rates ranging from 125 to 500 fps. The surface tension of SA/PEO/EtOH solutions was captured by a drop shape analyzer (DSA 30S, KRUSS, Hamburg, Germany).

### 2.4. Blow Spinning of SA/PEO/EtOH Solutions

SA/PEO/EtOH fibrous membranes were fabricated by a home-made blow spinning device, which is composed of a concentric nozzle, air source, infrared lamp, and collection roller. The SA/PEO/EtOH spinning solutions were extruded through a needle with an inner diameter of 860 µm and an outer diameter of 1260 µm via a syringe pump at a flow rate of 5 µL/min. These diameters and flow rate were selected to ensure the formation of a continuous jet and avoid clogging. The extruded solution was rapidly stretched by compressed air at a specific pressure ranging from 0.055 to 0.135 MPa to form a jet, while the solvent was evaporated by infrared lamps before the filaments reached the collection roller. The dried filaments were subsequently collected on a roller with a rotation speed of 19 r/min to form isotropic fibrous membranes. The spinning solutions and air pressures used for blow spinning and corresponding spinnability are shown in Table 2. The mechanical properties of the fibrous membranes were examined at room temperature using a commercial tensile tester (REGER, Shenzhen, China). All tests were conducted on rectangular membranes (25 mm × 5 mm) with an initial gauge length of 20 mm and a tensile rate of 10 mm/s. The thickness of each strip was measured with a thickness gauge, and the thickness adopted for calculation was the average of measurements at three different locations. The cross-sectional area of the sample was then calculated by multiplying the thickness by the width. Young’s modulus of the sample was calculated from the initial slope of the stress–strain curve with a strain below 5%. Five parallel tests were conducted on the sample. For morphological characterizations, the membrane samples were dried in a vacuum oven and cooled at room temperature for 24 h. The samples were then sputter-coated with gold and observed using a scanning electron microscope (ZEISS, Jena, Germany) at an acceleration voltage of 5 kV. The crosslinked SA-2.5-30-10 fibrous membrane was formed by soaking in calcium chloride solutions for 10 min and drying. The wetting processes and contact angles of water on the crosslinked membrane were conducted using a drop shape analyzer (DSA30S). The pore size distribution of the membrane was characterized by a pore size analyzer (TOPASPSM165, TOPAS, Germany).

### 2.5. Statistical Analysis

Rheological and mechanical measurements in this study were examined by three and five parallel tests, respectively. The reptation time, specific viscosity, extensional relaxation time, and mechanical parameters were shown in the figures with a standard deviation of the mean (*n* = 5).

## 3. Results and Discussion

### 3.1. The Effects of PEO and Ethanol on the Stretchability of SA-c_p_-f_PEO_-f_EtOH_ Solutions

**Figure 1:**
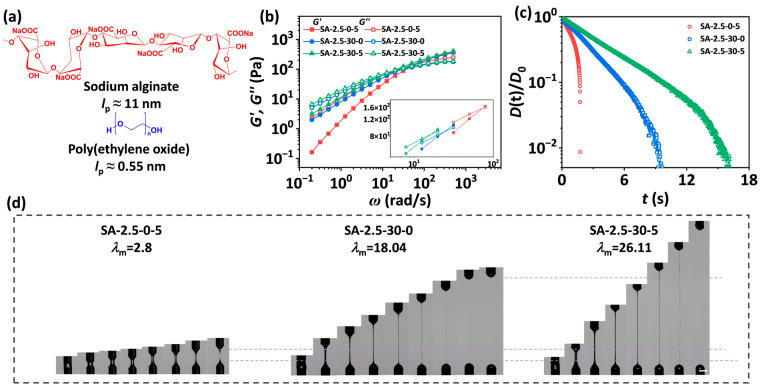
(**a**) The schematic diagram of the chemical structures of SA and PEO. (**b**) The storage (*G′*) and loss modulus (*G*″) of SA-2.5-0-5, SA-2.5-30-0, and SA-2.5-30-5 solutions as a function of angular frequency (*ω*). (**c**) Normalized midpoint diameter of liquid bridges as a function of time for the solutions. (**d**) The sequences of images captured by the high-speed camera show the stretch-to-rupture process of the solutions. The scale bar is 4 mm. To facilitate a clearer comparison of the maximum stretch ratio for three filaments, dashed lines were added to the figure.

The molecular structures of SA and PEO are presented in Figure 1a. Sodium alginate is a linear copolymer composed of (1,4)-linked β-D-mannuronate (M) blocks and α-L-guluronate (G) blocks. Its backbone contains hydrophilic groups, including carboxyl and hydroxyl groups. SA chains are semi-flexible, exhibiting a persistence length *l*_p_ of ~11 nm [35], while PEO chains are flexible with *l*_p_ of ~0.55 nm [36]. The oxygen atoms along the PEO backbone can form hydrogen bonds with water molecules. The effects of PEO and ethanol on the shear rheological behaviors and stretchability of SA solutions are elucidated (Figure 1b–d). With the addition of ethanol, the SA-2.5-0-5 solution reveals relatively low dynamic moduli with a reptation time (*τ*, the reciprocal of the crossover point of *G′* and *G*″) of 0.0136 s. The pinch-off time (*t*_p_) and maximum stretchability (*λ*_m_) of the solution are 1.76 s and 2.8, respectively, demonstrating that the incorporation of ethanol is not able to endow SA solutions with a high stretchability. On the other hand, *τ* of the SA solution increases to 0.0418 s with the addition of 30 wt.% PEO. The values of *t*_p_ and *λ*_m_ for the SA-2.5-30-0 solution are 9.62 s and 18.04, respectively, demonstrating PEO can drastically delay the pinch-off and increase the stretchability of SA solutions. Compared to SA-2.5-30-0 samples, the SA solution with the addition of both PEO and ethanol (SA-2.5-30-5) reveals further increased *τ* (0.0609 s), *t*_p_ (16.12 s), and *λ*_m_ (26.11). The rheological behaviors of the three solutions disclose that flexible polymers with high molecular weight and poorer solvents can synergistically delay the capillary-driven pinch-off and enhance the stretchability of semi-flexible SA solutions.

### 3.2. Shear Rheological Properties of SA-c_p_-f_PEO_-f_EtOH_ Solutions

**Figure 2:**
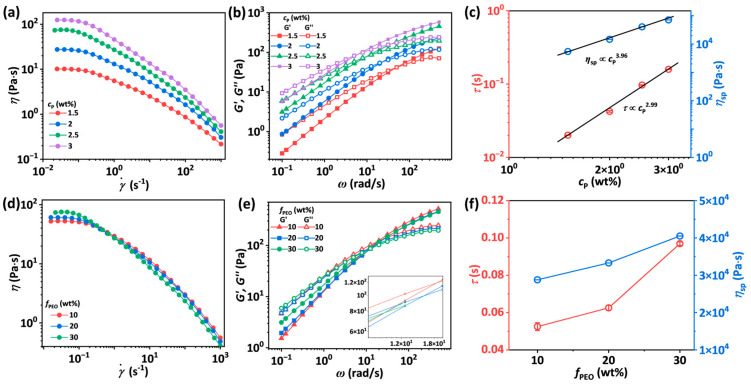
(**a**,**b**) Steady shear viscosity as a function of shear rate (**a**) and storage (*G′*) and loss (*G*″) modulus as a function of frequency (**b**) for SA-*c*_p_-30-10 solutions with *c*_p_ ranging from 1.5 wt.% to 3 wt.%. (**c**) Reptation time *τ* and specific viscosity *η*_sp_ as a function of *c*_p_ for SA-*c*_p_-30-10 solutions. The error bars represent the standard deviation of the mean (*n* = 5). (**d**,**e**) Steady shear viscosity as a function of shear rate (**d**) and storage (*G′*) and loss (*G*″) modulus as a function of frequency (**e**) for SA-2.5-*f*_PEO_-10 solutions with *f*_PEO_ ranging from 10 wt.% to 30 wt.%. (**f**) Reptation time *τ* and specific viscosity *η*_sp_ as a function of PEO content for SA-2.5-*f*_PEO_-10 solutions. The error bars represent the standard deviation of the mean (*n* = 5).

At fixed *f*_PEO_ and *f*_EtOH_, the curves of steady shear viscosity versus shear rate for SA-*c*_p_-30-10 solutions show a plateau at low shear rates and a shear-thinning characteristic at high shear rates (Figure 2a). As *c*_p_ increases, the zero shear viscosity *η*_0_ rises dramatically, and the shear-thinning behavior becomes more prominent, manifesting in a smaller non-Newtonian index *n*_s_ (Table S1). Figure 2b shows that *G′* and *G*″ of the solutions both increase with angular frequency (*ω*) and exhibit a crossover point, indicating a solid-like behavior at high frequencies and a liquid-like behavior at low frequencies. The specific viscosity *η*_sp_ = (*η*_0_ − *η*_s_)/*η*_s_ and reptation time *τ* as a function of *c*_p_ are plotted in Figure 2c. These two values of the solutions increase with *c*_p_ because of the higher entanglement density. Both *η*_sp_ and *τ* exhibit power-law dependencies on *c*_p_, described as *η*_sp_ ∝ *c*_p_^3.96^ and *τ* ∝ *c*_p_^2.99^, respectively. The power-law exponent for *η*_sp_ versus *c*_p_ is close to that reported in the literature for concentrated polyelectrolyte solutions (15/4) [37,38].

At fixed *c*_p_ and *f*_EtOH_, the zero-shear viscosity of the solutions exhibits a slight increase with the increasing PEO mass fraction *f*_PEO_. A more prominent shear-thinning behavior is exhibited because PEO chains are more flexible compared to SA chains (Figure 2d). From the dynamic modulus spectra in Figure 2e, it is revealed that the variation in *f*_PEO_ imposes no significant effect on dynamic moduli in the high-frequency region. However, with increasing *f*_PEO_, the crossover point of *G′* and *G*″ moves toward lower *ω*. As *f*_PEO_ increases from 10 wt.% to 30 wt.%, *η*_sp_ rises from 28,794.1 Pa∙s to 40,528.4 Pa∙s, and *τ* increases from 0.05 s to 0.10 s (Figure 2f). This demonstrates that the incorporation of flexible PEO chains with ultra-high molecular weight mainly provides additional entanglements for semi-flexible SA chains with lower molecular weight, increasing the viscosity of the solutions and the reptation time of polymer chains.

**Figure 3:**
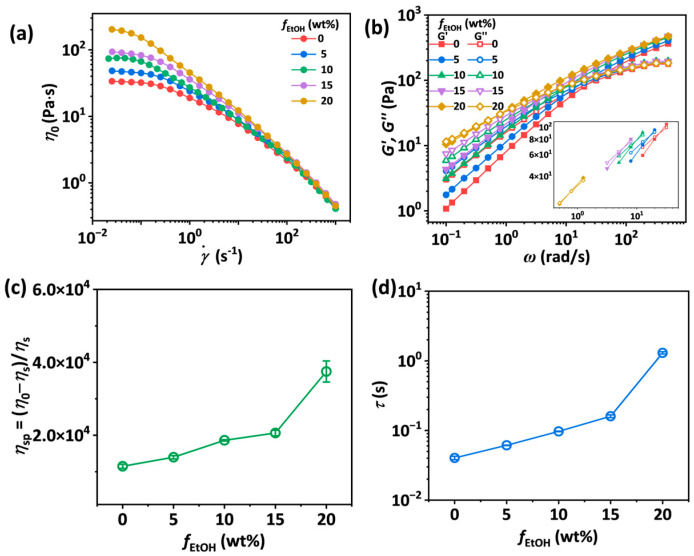
(**a**,**b**) Steady shear viscosity as a function of shear rate (**a**) and storage (*G′*) and loss (*G*″) modulus as a function of angular frequency (**b**) for SA-2.5-30-*f*_EtOH_ solutions with *f*_EtOH_ ranging from 0 to 20 wt.%. (**c**) Specific viscosity *η*_sp_ as a function of *f*_EtOH_ for SA-2.5-30-*f*_EtOH_ solutions. The error bars represent the standard deviation of the mean (*n* = 5). (**d**) Reptation time *τ* as a function of *f*_EtOH_ for SA-2.5-30-*f*_EtOH_ solutions.

To investigate the influence of the poor solvent effect of ethanol on the SA/PEO/EtOH solutions, steady shear viscosity and dynamic moduli of SA-2.5-30-*f*_EtOH_ solutions were measured. As shown in Figure 3a, *η*_0_ of SA-2.5-30-*f*_EtOH_ solutions increases with rising *f*_EtOH_. The value of *n*_s_ representing the shear-thinning behavior first decreases from 0.295 to 0.217 and then reaches equilibrium with further increase in *f*_EtOH_ (Table S1). The value of *n*_s_ can be obtained by fitting the data from shear-thinning regions using Equation (1). The values of *G′* and *G*″ of the solutions also increase with *f*_EtOH_, along with the crossover point shifting toward lower *ω*, especially when *f*_EtOH_ is beyond 15 wt.% (Figure 3b). As plotted in Figure 3c,d, *η*_sp_ and *τ* of the solutions increase from 11,484.3 Pa∙s to 37,480.1 Pa∙s and 0.04 s to 1.30 s, respectively, as *f*_EtOH_ rises from 0 to 20 wt.%, because ethanol acts as a poorer solvent than water for SA and PEO. Adding ethanol to SA/PEO solutions weakens the hydrogen bonds between water and SA as well as water and PEO. Therefore, fewer water molecules exist between polymer chains, bridging polymer chains through hydrogen bonding [39]. It should be noted that since SA chains possess more hydrogen bond sites, the poor solvent effect of ethanol exerted on SA chains is more significant than that on PEO chains.

### 3.3. Extensional Rheological Properties of SA-c_p_-f_PEO_-f_EtOH_ Solutions

**Figure 4:**
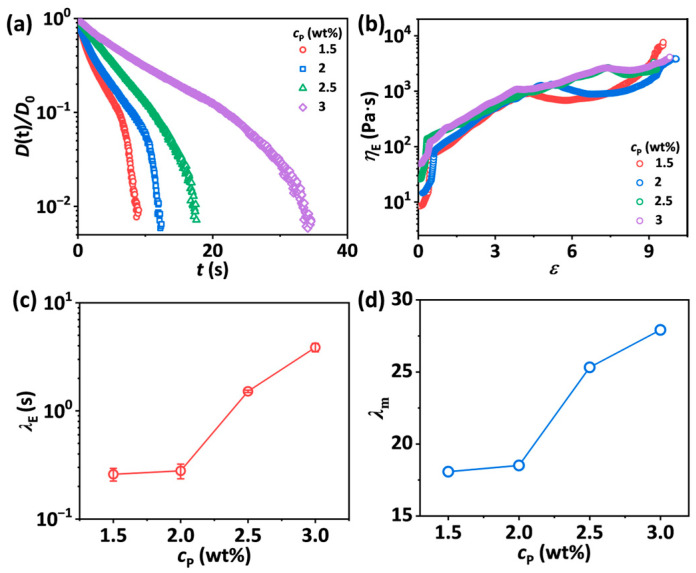
(**a**) Normalized midpoint diameter of liquid bridges as a function of time for SA-*c*_p_-30-10 solutions with *c*_p_ ranging from 1.5 wt.% to 3 wt.%. (**b**) The apparent extensional viscosity *η*_E_ as a function of Hencky strain for SA-*c*_p_-30-10 solutions. (**c**) Extensional relaxation time *λ*_E_ as a function of *c*_p_ for SA-*c*_p_-30-10 solutions. The error bars represent the standard deviation of the mean (*n* = 5). (**d**) The apparent maximum stretch ratio *λ*_m_ as a function of *c*_p_ for SA-*c*_p_-30-10 solutions.

Compared to shear rheological behaviors, the extensional rheological responses of polymer solutions at highly stretched states show more significant impacts on the morphology of spun fibers during blow spinning. First, the normalized mid-point diameter curves over time captured by CaBER for SA-*c*_p_-30-10 solutions are plotted on a semi-log y-axis in Figure 4a. When *c*_p_ ≤ 2 wt.%, the normalized diameter decay curves show a transition from a viscous capillary (VC) behavior to an elasto-capillary (EC) behavior. At higher *c*_p_, the curves exhibit a transition from an EC response to a terminal visco-elasto-capillary (TVEC) response. It is shown that the pinch-off time (*t*_p_) of the stretched filament increases with *c*_p_, corresponding to the sequences of images captured by the high-speed camera (Figure S1). To further clarify the effects of the component of SA/PEO/EtOH solutions on extensional rheology behaviors, the apparent extensional viscosity *η*_E_ is calculated by Equation (4). Figure 4b shows that when *c*_p_ ≤ 2 wt.%, *η*_E_ initially increases with strain, reaches a plateau, and then increases rapidly. In contrast, when *c*_p_ > 2 wt.%, *η*_E_ initially increases with strain and then tends to level off, reaching a steady terminal extensional viscosity $\eta_{E}^{\infty }$. As shown in Figure 4c, *λ*_E_ ascertained from fitting increases from 0.26 s to 3.50 s as *c*_p_ increases from 1.5 wt.% to 3 wt.%, indicating the higher polymer concentration delays the chain relaxation after stretching. The fitting profiles are shown in Figure S2. Meanwhile, the apparent maximum stretch ratio *λ*_m_ obtained from the stretch-to-rupture processes increases from 18.08 to 27.92 with rising *c*_p_ due to the decreased surface tension and enhanced entanglements that resist capillary-driven rupture of the filaments (Figure 4d and Figure S3).

**Figure 5:**
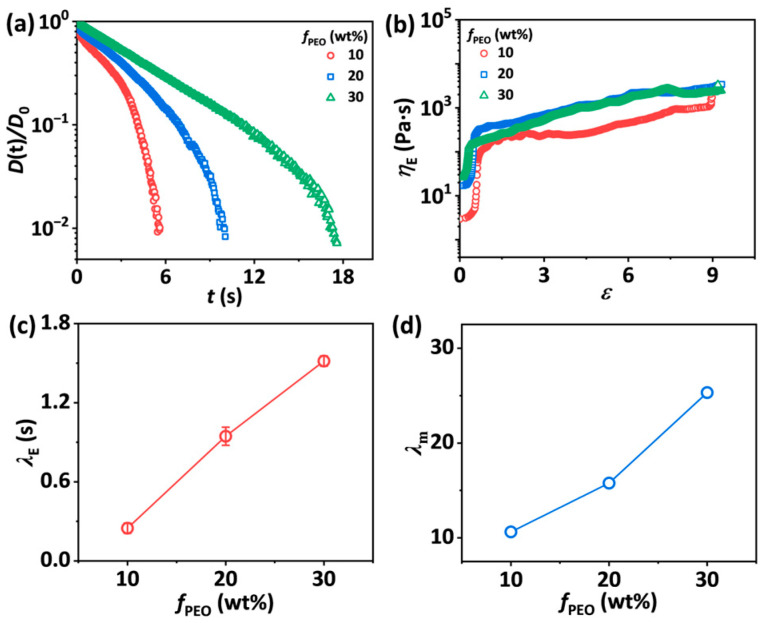
(**a**) Normalized midpoint diameter of liquid bridges as a function of time for SA-2.5-*f*_PEO_-10 solutions with *f*_PEO_ ranging from 10 wt.% to 30 wt.%. (**b**) The apparent extensional viscosity *η*_E_ as a function of Hencky strain for SA-2.5-*f*_PEO_-10 solutions. (**c**) Extensional relaxation time *λ*_E_ as a function of *f*_PEO_ for SA-2.5-*f*_PEO_-10 solutions. The error bars represent the standard deviation of the mean (*n* = 5). (**d**) The apparent maximum stretch ratio *λ*_m_ as a function of *f*_PEO_ for SA-2.5-*f*_PEO_-10 solutions.

At fixed *c*_p_ and *f*_EtOH_, the normalized diameter evolution curves over time of SA-2.5-*f*_PEO_-10 solutions are shown in Figure 5a and fitted in Figure S4. When *f*_PEO_ is 10 wt.%, a transition from VC to EC regime is observed in the curve. As *f*_PEO_ is above 10 wt.%, the VC regime disappears, and a transition from EC to TVEC regime emerges. Figure 5b shows that the apparent extensional viscosity *η*_E_ of SA-2.5-20-10 and SA-2.5-30-10 solutions increases with increasing Hencky strain, and then levels off. The value of *λ*_E_ obtained by fitting the diameter decay curves exhibits an approximately linear dependence on *f*_PEO_ (Figure 5c). As *f*_PEO_ increases from 10 wt.% to 30 wt.%, *λ*_E_ rises from 0.21 s to 1.52 s (Figure 5c). The value of the maximum stretch ratio *λ*_m_ also shows a prominent increase from 10.63 to 25.32 with increasing *f*_PEO_ (Figure 5d and Figure S5). Unlike shear rheological behaviors, the effect of *f*_PEO_ on the extensional rheological behaviors of the solutions is mainly attributed to the larger intrinsic extensibility (the ratio of the contour length to the mean square end-to-end distance of polymer chains that is equal to *N*_k_^1−*ν*^, where *N*_k_ is the number of Kuhn segments in the chain and *ν* is the solvent quality exponent) of PEO chains than that of SA chains, leading to longer extensional relaxation time and larger maximum stretch ratio.

**Figure 6:**
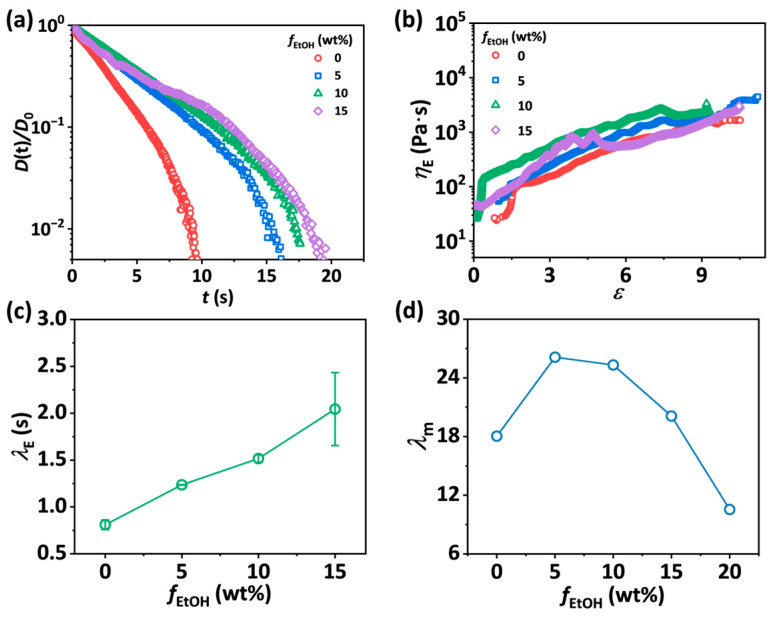
(**a**) Normalized midpoint diameter of liquid bridges as a function of time for SA-2.5-30-*f*_EtOH_ solutions with *f*_EtOH_ ranging from 0 to 15 wt.%. (**b**) The apparent extensional viscosity *η*_E_ as a function of Hencky strain of SA-2.5-30-*f*_EtOH_ solutions. (**c**) Extensional relaxation time *λ*_E_ as a function of *f*_EtOH_ s for SA-2.5-30-*f*_EtOH_ solutions. The error bars represent the standard deviation of the mean (*n* = 5). (**d**) The apparent maximum stretch ratio *λ*_m_ as a function of *f*_EtOH_ for SA-2.5-30-*f*_EtOH_ solutions.

Furthermore, the normalized diameter evolution curves over time of SA/PEO/EtOH solutions with various *f*_EtOH_ are plotted on a semi-log y axis in Figure 6a. A transition from the EC regime to the TVEC regime is exhibited in the curves of the solutions. As shown in Figure 6b, the apparent extensional viscosity *η*_E_ increases with Hencky strain *ε* and then levels off except for the SA-2.5-30-15 solution. The sequences of images illustrating the capillary-driven thinning processes of SA-2.5-30-*f*_EtOH_ solutions are presented in Figure S6. Note that the filament of SA-2.5-30-20 solutions becomes non-homogeneous during thinning due to the more solid-like property of the solution. Thus, the normalized diameter evolution curve of the solution fluctuates over time and is not included in Figure 6. The value of *λ*_E_ ascertained from fitting the curves by Equations (2) and (3) (Figure S7) shows a monotonic increase from 0.81 s to 2.04 s with *f*_EtOH_, demonstrating the enhanced hydrogen bonding between polymer chains via water molecules delays the relaxation of polymer chains after stretching (Figure 6c). To eliminate the effect of extensional viscosity of the solvents, a specific terminal extensional viscosity $\eta_{sp}^{\infty }$ defined as ($\eta_{E}^{\infty }$ − 3*η*_s_)/3*η*_s_ is calculated. As shown in Table S3, the values of $\eta_{sp}^{\infty }$ first increases with *f*_EtOH_ until reaching a maximum at *f*_EtOH_ = 5 wt.%, and then decreases. A specific terminal Trouton ratios *Tr*^∞^ is defined as ${{Tr}^{\infty }=(\eta }_{E}^{\infty }-3\eta_{s})/(\eta_{0}-\eta_{s}$) for the solutions [40]. Since *Tr*^∞^ is equal to 2(1 − *η*_s_/*η*_0_) *L*^2^, where *η*_s_ is the solvent viscosity and *L* is equal to chain extensibility, *L* of the solutions also shows a first increasing and then decreasing trend with *f*_EtOH_. The above analysis reveals that the slightly enhanced hydrogen bonding promotes the extension of chains during stretch, while further stronger interactions could hamper the full extension of chains and lower chain extensibility. The apparent maximum stretch ratio *λ*_m_ of the solutions is obtained from the stretch-to-rupture processes (Figure S8). As shown in Figure 6d, *λ*_m_ exhibits a maximum value of 26.11 at *f*_EtOH_ is equal to 5 wt.%, which is 44.7% larger than that of SA/PEO solutions in the absence of ethanol due to the higher chain extensibility.

### 3.4. Blow Spinning of SA-c_p_-f_PEO_-f_EtOH_ Solutions

**Figure 7:**
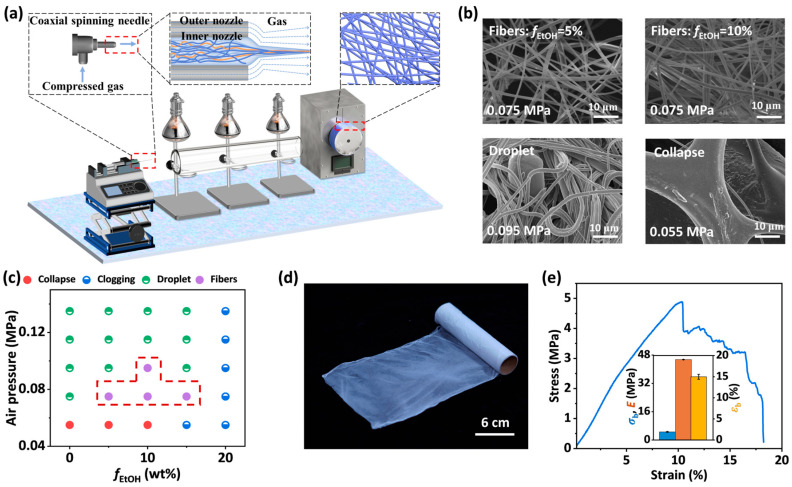
(**a**) Schematic of blow spinning procedure. (**b**) SEM images of typical micro-fibrous membranes fabricated by SA/PEO/EtOH solutions with different *f*_EtOH_ and under different air pressure. (**c**) Spinning diagram of SA-2.5-30-*f*_EtOH_ solutions with different air pressure and *f*_EtOH_. The region enclosed by the dashed box in the figure corresponds to the processing conditions that yield good spinnability. (**d**) Photo of a micro-fibrous membrane obtained by blow spinning. (**e**) The stress–strain curve and corresponding mechanical properties of the micro-fibrous membrane. The error bars represent the standard deviation of the mean (*n* = 5).

SA-2.5-30-*f*_EtOH_ solutions were adopted for blow spinning using various spinning parameters. The polymer solutions were extruded through the inner nozzle of a coaxial needle while high-velocity air was supplied through the outer nozzle to stretch the solutions and form thin filaments. With the evaporation of solvents by infrared lamps, dried filaments are randomly deposited onto a collector, forming a non-woven fibrous membrane (Figure 7a). With the absence of ethanol, the poor stretchability of the solutions results in the rupture of filaments during spinning, and only droplets were observed on the collector. SEM images of fibrous membranes fabricated by SA-2.5-30-*f*_EtOH_ solutions with *f*_EtOH_ ranging from 5 wt.% to 15 wt.% exhibit homogeneous networks composed of fibers with a diameter of ~1 µm (Figure 7b and Figure S9). The diameter distribution of the fibers is shown in Figure S10. The further increase in the ethanol concentration to 20 wt.% leads to a significant increase in solution viscosity, causing needle clogging.

Furthermore, air pressure also plays a critical role in the morphology of the membranes. As shown in Figure 7b and Figure S11, insufficient air pressure fails to provide adequate stretch to the solutions. Thus, the fibers are not completely dried when arriving at the collector, resulting in the collapse of fibers. However, excessively high pressure causes the filaments to rupture during stretch and eventually form droplets. A spinning diagram of SA-2.5-30-*f*_EtOH_ solutions with different air pressure and *f*_EtOH_ is shown in Figure 7c. After optimizing the ethanol concentration and air pressure, the SA-2.5-30-10 solution was selected for blow spinning at an air pressure of 0.075 MPa. Note that the SA-2.5-30-10 solution is selected here because the solution has a higher solvent evaporation rate due to the higher concentration of ethanol, which is more suitable for the fabrication of membranes with a large thickness. Alginate-based fibrous membranes with ~30 cm in length and ~12 cm in width were successfully fabricated (Figure 7d). The optical micrograph of the SA-2.5-30-10 fiber membrane further confirms the successful fabrication of an alginate-based fibrous membrane with uniform morphology (Figure S12). The membrane shows a tensile strength of 4.89 MPa, an elongation at break of 15.24%, and a Young’s modulus of 45.43 MPa (Figure 7e). Moreover, the feasibility of alginate-based fibrous membranes as functional membranes is verified. The SA-2.5-30-10 fibrous membrane is crosslinked in calcium chloride solutions for 10 min and dried. The chelation between sodium alginate and calcium ions ensures the membrane is insoluble in water. As soon as the drop of water contacts the membrane, it is immediately absorbed by the membrane (Figure S13). The water contact angle of the crosslinked membrane is about 34°, demonstrating the hydrophilicity of the membrane. The pore size distribution of the fibrous membrane is also characterized to reveal its capacity for filtering particle pollution in the air. The result shows that the average pore diameter of the membrane is ~280 nm, implying the membrane can filter substances larger than this critical size (Figure S14). These properties make the alginate-based membrane a promising candidate for filtration and adsorption in biomedical applications.

## 4. Conclusions

In this paper, we systematically investigate the shear and extensional rheological behaviors of a series of sodium alginate/polyethylene oxide solutions in solvent mixtures of water and ethanol. It is found that the increases in the total polymer concentration and PEO mass fraction both increase the zero-shear viscosity, extensional relaxation time, and apparent maximum stretch ratio of the solutions, indicating flexible polymers with high molecular weight effectively increase the entanglement density and thus improve the stretchability of the solutions. The rise in the ethanol concentration from 0 to 20 wt.% increases the specific shear viscosity and extensional relaxation time from 11,484 to 37,480 and 0.81 s to 2.04 s, respectively, mainly due to the enhanced polymer–polymer interactions. The terminal Trouton ratio and apparent maximum stretch ratio both show a maximum value at the ethanol concentration of 5 wt.%, demonstrating that an appropriate amount of ethanol as a co-solvent increases polymer chain extensibility. By optimizing the spinning solution composition and processing parameters, alginate-based micro-fibrous membranes with a tensile strength of 4.89 MPa, an elongation at break of 15.24%, and a Young’s modulus of 45.43 MPa are successfully prepared via blow spinning. This work proposes a facile strategy to fabricate micro-fibrous membranes based on sodium alginate by modulating solvent composition, paving the way for the design of advanced polysaccharide membranes with specific functions and applications.

## Figures and Tables

**Table 1:** The compositions of SA-*c*_p_-*f*_PEO_-*f*_EtOH_ solutions.

SA-*c*_p_-*f*_PEO_-*f*_EtOH_	*c*_p_ (wt.%)	*f*_PEO_ (wt.%)	*f*_EtOH_ (wt.%)
SA-1.5-30-10	1.5	30	10
SA-2-30-10	2.0	30	10
SA-2.5-30-10	2.5	30	10
SA-3-30-10	3.0	30	10
SA-2.5-10-10	2.5	10	10
SA-2.5-20-10	2.5	20	10
SA-2.5-30-0	2.5	30	0
SA-2.5-30-5	2.5	30	5
SA-2.5-30-15	2.5	30	15
SA-2.5-30-20	2.5	30	20

**Table 2:** Processing parameters for blow spinning.

Spinning Solution	Air Pressure (MPa)
SA-2.5-30-0	0.055
SA-2.5-30-5	0.075
SA-2.5-30-10	0.095
SA-2.5-30-15	0.115
SA-2.5-30-20	0.135

## Data Availability

The original contributions presented in this study are included in the article/Supplementary Materials. Further inquiries can be directed to the corresponding author.

## Supplementary Materials

The following supporting information can be downloaded at: https://www.mdpi.com/article/10.3390/ma18245491/s1, Figure S1: Sequences of images captured by the high-speed camera to show the capillary-driven thinning processes of SA-cp-30-10 solutions (a) and SA-2.5-fPEO-10 solutions (b). The scale bar is 6 mm; Figure S2: Fitting profiles of SA-cp-30-10 solutions with cp ranging from 1.5 wt.% to 3 wt.%; Figure S3: The sequences of images captured by the high-speed camera to show the stretch-to-rupture processes of SA-cp-30-10 solutions with cp ranging from 1.5 wt.% to 3 wt.%. The scale bar is 4 mm; Figure S4: Fitting profiles of SA-2.5-fPEO-10 solutions with fPEO ranging from 10 wt.% to 30 wt.%; Figure S5: The sequences of images captured by the high-speed camera to show the stretch-to-rupture processes of SA-2.5-fPEO-10 solutions with fPEO ranging from 10 wt.% to 30 wt.%. The scale bar is 4 mm; Figure S6: Sequences of images captured by the high-speed camera to show the capillary-driven thinning processes of SA-2.5-30-fEtOH. The scale bar is 6 mm; Figure S7: Fitting profiles of SA-2.5-30-fEtOH solutions with fEtOH ranging from 0 to 15 wt.%; Figure S8: The sequences of images captured by the high-speed camera to show the stretch-to-rupture processes of SA-2.5-30-fEtOH solutions with fEtOH ranging from 0 to 20 wt.%. The scale bar is 4 mm; Figure S9: SEM images of blowing-spinning fibrous membranes (SA-2.5-30-fEtOH) when the air pressure is 0.075 MPa; Figure S10: Statistical diagram of fiber diameter distribution for SA-2.5-30-5 (a) and SA-2.5-30-10 (b) fibrous membranes when the air pressure is 0.075 MPa. Figure S11: SEM images of blowing-spinning fibrous membranes (SA-2.5-30-10) under different air pressures; Figure S12: The microscopic image of SA-2.5-30-10 fibrous membrane; Figure S13: Photo of the surface wettability behavior for water of the cross-linked SA-2.5-30-10 fibrous membranes; Figure S14: Pore size distribution of SA-2.5-30-10 fibrous membranes; Table S1: Non-Newtonian index ns of SA-cp-fPEO-fEtOH solutions; Table S2: Surface tension and initial filament diameter of SA-cp-fPEO-fEtOH solutions; Table S3: Fitting parameters of SA-*c*_p_-*f*_PEO_-*f*_EtOH_ solutions.

## References

[1] Gamage A., Thiviya P., Liyanapathiranage A., Wasana M.L.D., Jayakodi Y., Bandara A., Manamperi A., Dassanayake R.S., Evon P., Merah O. (2024). Polysaccharide-Based Bioplastics: Eco-Friendly and Sustainable Solutions for Packaging. J. Compos. Sci..

[2] Hammi N., Wronska N., Katir N., Lisowska K., Marcotte N., Cacciaguerra T., Bryszewska M., El Kadib A. (2019). Supramolecular Chemistry-Driven Preparation of Nanostructured, Transformable, and Biologically Active Chitosan-Clustered Single, Binary, and Ternary Metal Oxide Bioplastics. ACS Appl. Bio Mater..

[3] Kajla P., Chaudhary V., Dewan A., Bangar S.P., Ramniwas S., Rustagi S., Pandiselvam R. (2024). Seaweed-Based Biopolymers for Food Packaging: A Sustainable Approach for a Cleaner Tomorrow. Int. J. Biol. Macromol..

[4] Li X., Wang S., Sun Z., Gao M., Li Q., Qin M. (2024). Study on Enteromorpha Polysaccharide/Konjac Glucomannan Mulch Films with Biochar as a Fertilizer Carrier. ACS Appl. Polym. Mater..

[5] Wang Z., Xu C., Qi L., Chen C. (2024). Chemical Modification of Polysaccharides for Sustainable Bioplastics. Trends Chem..

[6] Dou X., Wang Q., Li Z., Ju J., Wang S., Hao L., Sui K., Xia Y., Tan Y. (2019). Seaweed-Derived Electrospun Nanofibrous Membranes for Ultrahigh Protein Adsorption. Adv. Funct. Mater..

[7] Gong X., Yang D., Wang N., Sun S., Nie J., Ma G. (2020). Polyethylenimine Grafted Chitosan Nanofiber Membrane as Adsorbent for Selective Elimination of Anionic Dyes. Fiber. Polym..

[8] Zhao X., Wang X., Lou T. (2022). Simultaneous Adsorption for Cationic and Anionic Dyes Using Chitosan/Electrospun Sodium Alginate Nanofiber Composite Sponges. Carbohydr. Polym..

[9] Zhu F., Zheng Y.M., Zhang B.G., Dai Y.R. (2021). A Critical Review on the Electrospun Nanofibrous Membranes for the Adsorption of Heavy Metals in Water Treatment. J. Hazard. Mater..

[10] Fijoł N., Aguilar-Sánchez A., Ruiz-Caldas M.-X., Redlinger-Pohn J., Mautner A., Mathew A.P. (2023). 3D Printed Polylactic Acid (PLA) Filters Reinforced with Polysaccharide Nanofibers for Metal Ions Capture and Microplastics Separation from Water. Chem. Eng. J..

[11] Sepahvand S., Kargarzadeh H., Jonoobi M., Ashori A., Ismaeilimoghadam S., Varghese R.T., Chirayl C.J., Azimi B., Danti S. (2023). Recent Developments in Nanocellulose-Based Aerogels as Air Filters: A Review. Int. J. Biol. Macromol..

[12] Zhang L., Li L., Wang L., Nie J., Ma G. (2020). Multilayer Electrospun Nanofibrous Membranes with Antibacterial Property for Air Filtration. Appl. Surf. Sci..

[13] He H., Wang Y., Yu Z., Liu J., Zhao Y., Ke Y. (2021). Ecofriendly Flame-Retardant Composite Aerogel Derived from Polysaccharide: Preparation, Flammability, Thermal Kinetics, and Mechanism. Carbohydr. Polym..

[14] Mu J., Chen X., Luo Z., Hui Z., Zhuo C., Zou F., Zhang S., Li H., Jian X. (2025). Fabrication of Strong and Thermally Insulated Sodium Alginate Aerogels via a Salt-Regulated Freeze-Casting Strategy. ACS Appl. Polym. Mater..

[15] Song M., Jiang J., Qin H., Ren X., Jiang F. (2020). Flexible and Super Thermal Insulating Cellulose Nanofibril/Emulsion Composite Aerogel with Quasi-Closed Pores. ACS Appl. Mater. Interfaces.

[16] Latonen R.M., Cabrera J.A.W., Lund S., Kosourov S., Vajravel S., Boeva Z., Wang X., Xu C., Allahverdiyeva Y. (2021). Electrospinning of Electroconductive Water-Resistant Nanofibers of PEDOT-PSS, Cellulose Nanofibrils and PEO: Fabrication, Characterization, and Cytocompatibility. ACS Appl. Bio Mater..

[17] Pereao O., Uche C., Bublikov P.S., Bode-Aluko C., Rossouw A., Vinogradov I.I., Nechaev A.N., Opeolu B., Petrik L. (2021). Chitosan/PEO Nanofibers Electrospun on Metallized Track-Etched Membranes: Fabrication and Characterization. Mater. Today Chem..

[18] Wongkanya R., Chuysinuan P., Pengsuk C., Techasakul S., Lirdprapamongkol K., Svasti J., Nooeaid P. (2017). Electrospinning of Alginate/Soy Protein Isolated Nanofibers and Their Release Characteristics for Biomedical Applications. J. Sci..

[19] Mengistu Lemma S., Bossard F., Rinaudo M. (2016). Preparation of Pure and Stable Chitosan Nanofibers by Electrospinning in the Presence of Poly (ethylene oxide). Int. J. Mol. Sci..

[20] Varnaite-Zuravliova S., Savest N., Baltusnikaite-Guzaitiene J., Abraitiene A., Krumme A. (2023). The Investigation of the Production of Salt-Added Polyethylene Oxide/Chitosan Nanofibers. Materials.

[21] Cestari M., Caldas B.S., Fonseca D.P., Balbinot R.B., Lazarin-Bidóia D., Otsuka I., Nakamura C.V., Borsali R., Muniz E.C. (2022). Silk Fibroin Nanofibers Containing Chondroitin Sulfate and Silver Sulfadiazine for Wound Healing Treatment. J. Drug Deliv. Sci. Technol..

[22] Gao T., Guan G., Wang X., Lou T. (2022). Electrospun Molecularly Imprinted Sodium Alginate/Polyethylene Oxide Nanofibrous Membranes for Selective Adsorption of Methylene Blue. Int. J. Biol. Macromol..

[23] Ahn Y., Hu D.H., Hong J.H., Lee S.H., Kim H.J., Kim H. (2012). Effect of Co-Solvent on the Spinnability and Properties of Electrospun Cellulose Nanofiber. Carbohydr. Polym..

[24] Vu T.H.N., Morozkina S.N., Olekhnovich R.O., Podshivalov A.V., Uspenskaya M.V. (2024). Study on Fabrication and Properties of Polyvinyl Alcohol/Chitosan Nanofibers Created from Aqueous Solution with Acetic Acid and Ethanol by the Electrospinning Method. Polymers.

[25] Vats S., Honaker L.W., Frey M.W., Basoli F., Lagerwall J.P.F. (2021). Electrospinning Ethanol–Water Solutions of Poly(Acrylic Acid): Nonlinear Viscosity Variations and Dynamic Taylor Cone Behavior. Macromol. Mater. Eng..

[26] Zheng J., Van der Meeren P., Sun W. (2023). New Insights into Protein–Polysaccharide Complex Coacervation: Dynamics, Molecular Parameters, and Applications. Aggregate.

[27] Dinic J., Sharma V. (2019). Macromolecular relaxation, strain, and extensibility determine elastocapillary thinning and extensional viscosity of polymer solutions. Proc. Natl. Acad. Sci. USA.

[28] Haward S.J., Sharma V., Butts C.P., McKinley G.H., Rahatekar S.S. (2012). Shear and extensional rheology of cellulose/ionic liquid solutions. Biomacromolecules.

[29] Jimenez L.N., Dinic J., Parsi N., Sharma V. (2018). Extensional Relaxation Time, Pinch-Off Dynamics, and Printability of Semidilute Polyelectrolyte Solutions. Macromolecules.

[30] Jimenez L.N., Martínez Narváez C.D.V., Sharma V. (2020). Capillary breakup and extensional rheology response of food thickener cellulose gum (NaCMC) in salt-free and excess salt solutions. Phys. Fluids.

[31] Martínez Narváez C.D.V., Dinic J., Lu X., Wang C., Rock R., Sun H., Sharma V. (2021). Rheology and Pinching Dynamics of Associative Polysaccharide Solutions. Macromolecules.

[32] Du W., Fu T., Zhang Q., Zhu C., Ma Y., Li H.Z. (2016). Breakup Dynamics for Droplet Formation in a Flow-Focusing Device: Rupture Position of Viscoelastic Thread from Matrix. Chem. Eng. Sci..

[33] Muto M.T.Y., Tamano S. (2024). Simultaneous Measurement of Extensional Stress and Flow Birefringence Field for Uniaxially Extending Worm-Like Micellar Solutions. arXiv.

[34] Anna S.L., McKinley G.H. (2001). Elasto-Capillary Thinning and Breakup of Model Elastic Liquids. J. Rheol..

[35] Banerjee A., De R., Das B. (2022). Hydrodynamic and Conformational Characterization of Aqueous Sodium Alginate Solutions with Varying Salinity. Carbohydr. Polym..

[36] Dinic J., Sharma V. (2020). Flexibility, Extensibility, and Ratio of Kuhn Length to Packing Length Govern the Pinching Dynamics, Coil-Stretch Transition, and Rheology of Polymer Solutions. Macromolecules.

[37] Colby S.D.R.H. (2008). Solution Rheology of a Strongly Charged Polyelectrolyte in Good Solvent. Macromolecules.

[38] Dou S., Colby R.H. (2006). Charge Density Effects in Salt-Free Polyelectrolyte Solution Rheology. J. Polym. Sci. Part B Polym. Phys..

[39] Zhou Q., Chng C.-P., Zhao Y., Wang Y., Xu H., Huo Y., Huang C. (2024). Ethanol-Induced Gelation Enables Direct Three-Dimensional Printing of Sodium Alginate Hydrogel. Mater. Des..

[40] Jimenez L.N., Martínez Narváez C.D.V., Sharma V. (2022). Solvent Properties Influence the Rheology and Pinching Dynamics of Polyelectrolyte Solutions: Thickening the Pot with Glycerol and Cellulose Gum. Macromolecules.

