# Supplementary material for: Study of Extensional Rheology Behavior of Sodium Alginate/Polyethylene Oxide Solutions for Blow Spinning
Source: Materials (Basel). 2025 Dec 5;18(24):5491. doi: 10.3390/ma18245491 (PMC12735334; doi:10.3390/ma18245491)
Supplement: Supplementary file 1 [file materials-18-05491-s001.zip › materials-3980008-supplementary.pdf]

Supporting Information

Study of Extensional Rheology Behavior of Sodium Alginate/Polyethylene Oxide Solutions for Blow Spinning

Biao Yang[1,†], Xue Wang[1,†], Cong Du[1,*]

[1]Shandong Key Laboratory of Renewable Membrane Materials, College of Materials Science and Engineering, Qingdao University, Qingdao 266071, China; yb19862511837@163.com (B.Y.); yawx128ve980@163.com (X.W.)

†They contributed equally to this work.

*Corresponding authors. *E-mail addresses*: cong.du@qdu.edu.cn (C. Du)

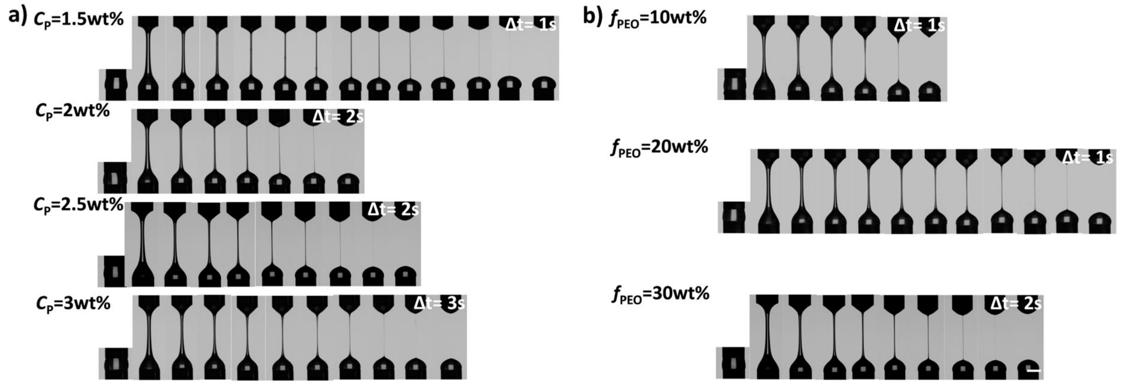

Figure S1. Sequences of images captured by the high-speed camera to show the capillary-driven thinning processes of SA-c_P-30-10 solutions (a) and SA-2.5-f_{PEO}-10 solutions (b). The scale bar is 6 mm.

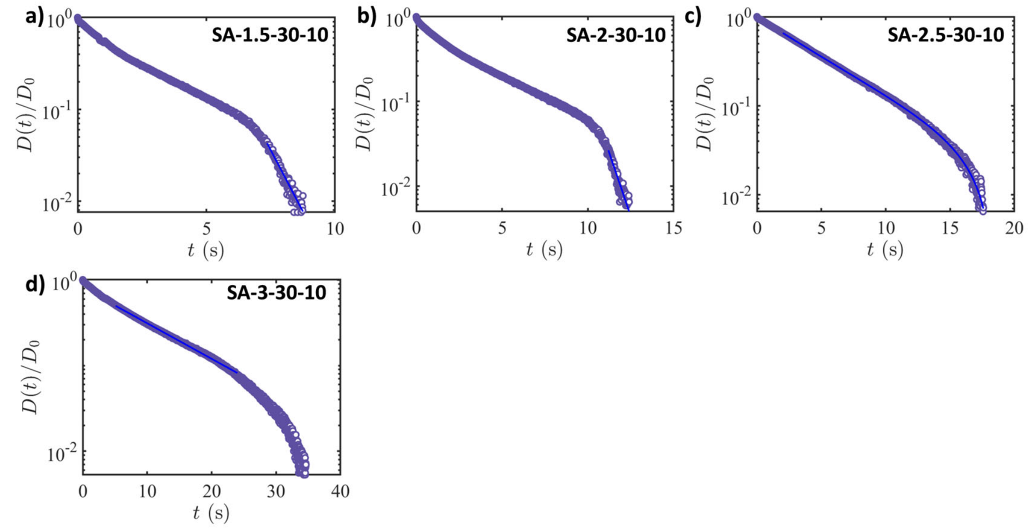

Figure S2. Fitting profiles of SA-c_P-30-10 solutions with c_P ranging from 1.5 wt.% to 3 wt.%.

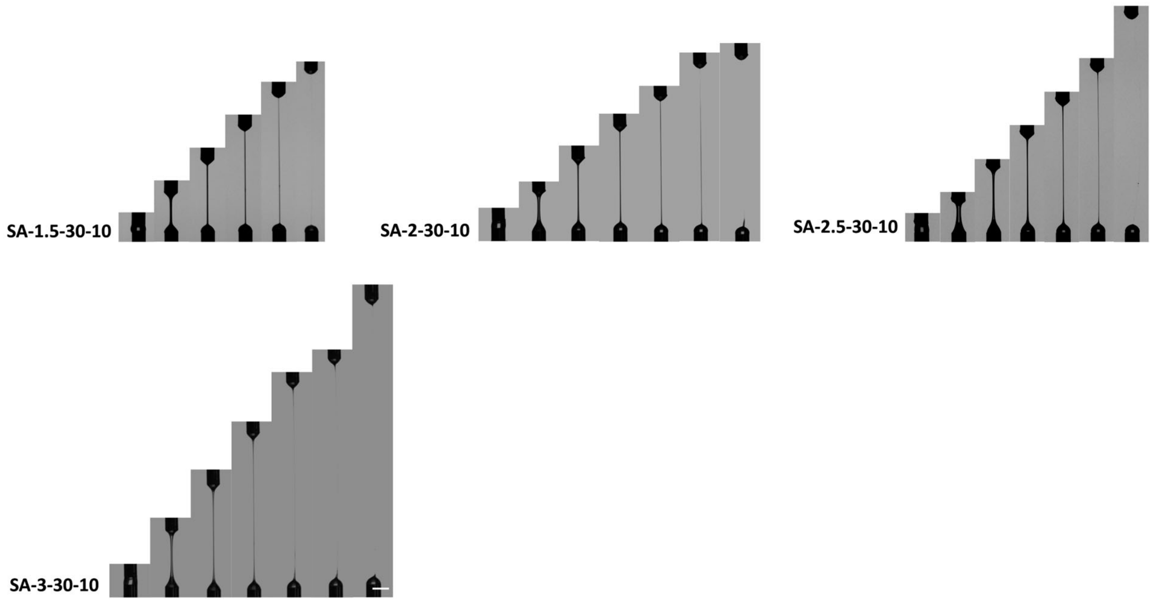

Figure S3. The sequences of images captured by the high-speed camera to show the stretch-to-rupture processes of SA-c_p-30-10 solutions with c_p ranging from 1.5 wt.% to 3 wt.%. The scale bar is 4 mm.

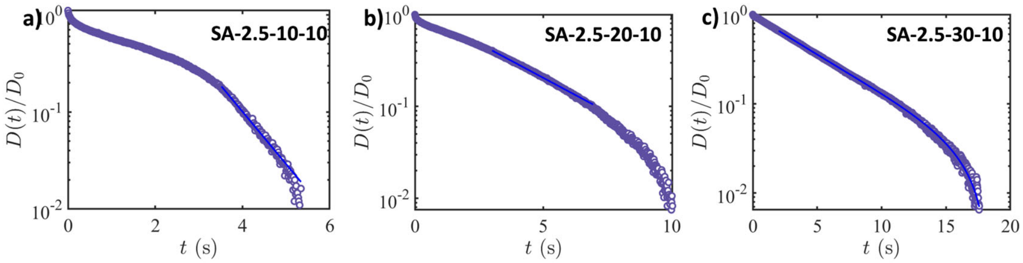

Figure S4. Fitting profiles of SA-2.5-f_{PEO}-10 solutions with f_{PEO} ranging from 10 wt.% to 30 wt.%.

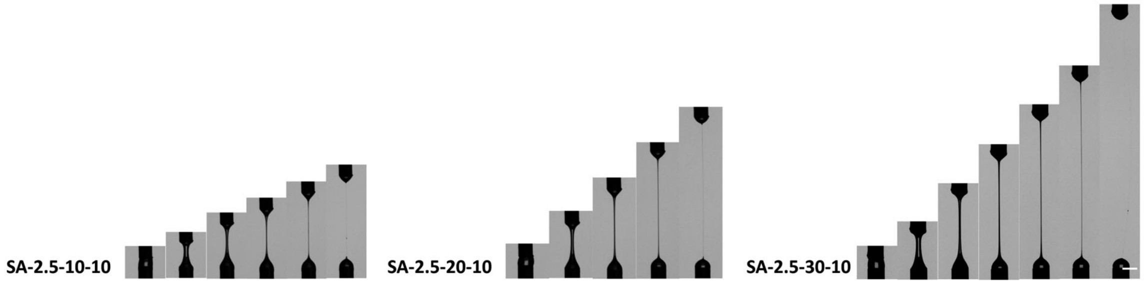

Figure S5. The sequences of images captured by the high-speed camera to show the stretch-to-rupture processes of SA-2.5-f_{PEO}-10 solutions with f_{PEO} ranging from 10 wt.% to 30 wt.%. The scale bar is 4 mm.

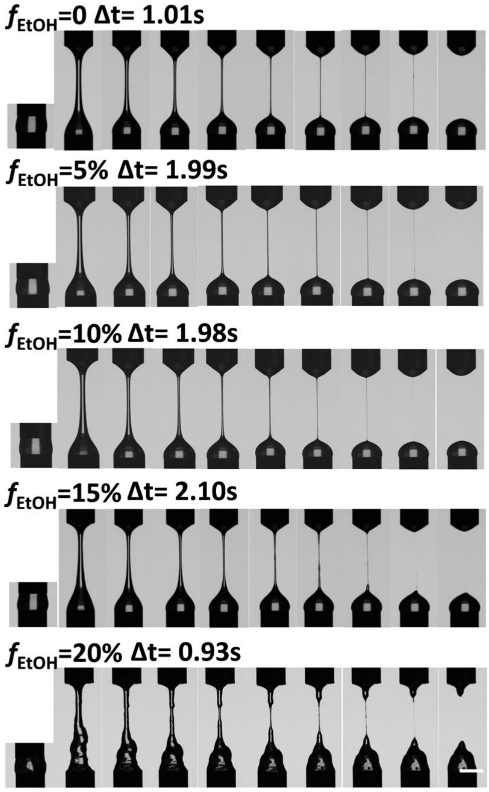

Figure S6. Sequences of images captured by the high-speed camera to show the capillary-driven thinning processes of SA-2.5-30-f_{EtOH}. The scale bar is 6 mm.

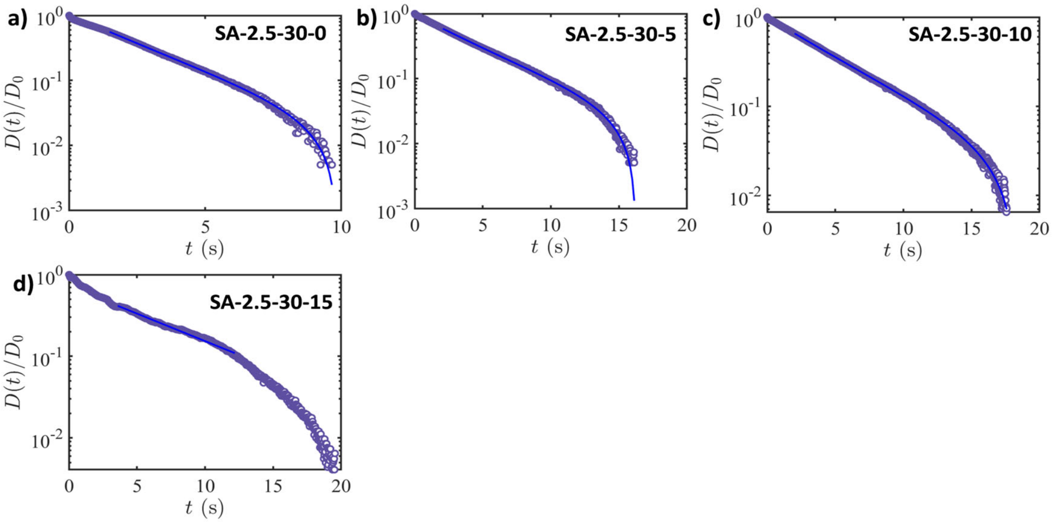

Figure S7. Fitting profiles of SA-2.5-30-f_{EtOH} solutions with f_{EtOH} ranging from 0 to 15 wt.%.

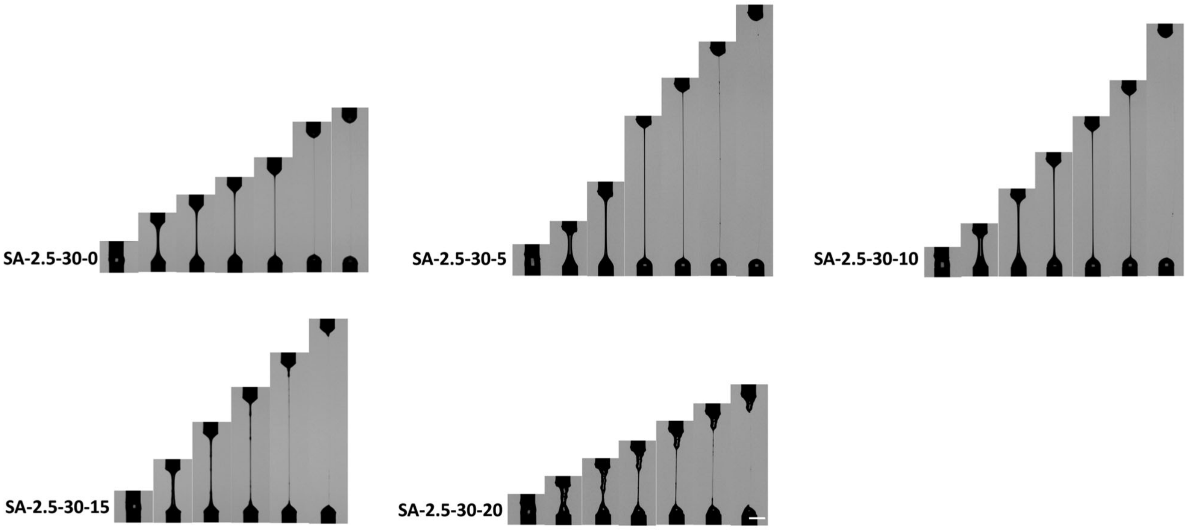

Figure S8. The sequences of images captured by the high-speed camera to show the stretch-to-rupture processes of SA-2.5-30-f_{EtOH} solutions with f_{EtOH} ranging from 0 to 20 wt.%. The scale bar is 4 mm.

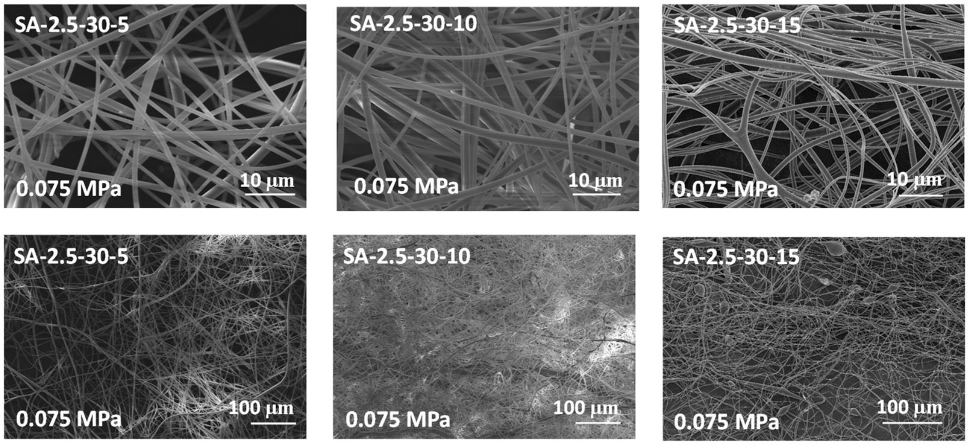

Figure S9. SEM images of blowing-spinning fibrous membranes (SA-2.5-30-f_{EtOH}) when the air pressure is 0.075 MPa.

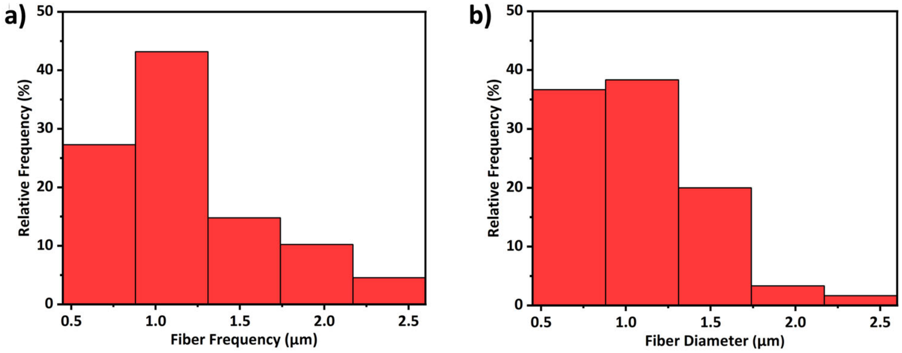

Figure S10. Statistical diagram of fiber diameter distribution for SA-2.5-30-5 (a) and SA-2.5-30-10 (b) fibrous membranes when the air pressure is 0.075 MPa.

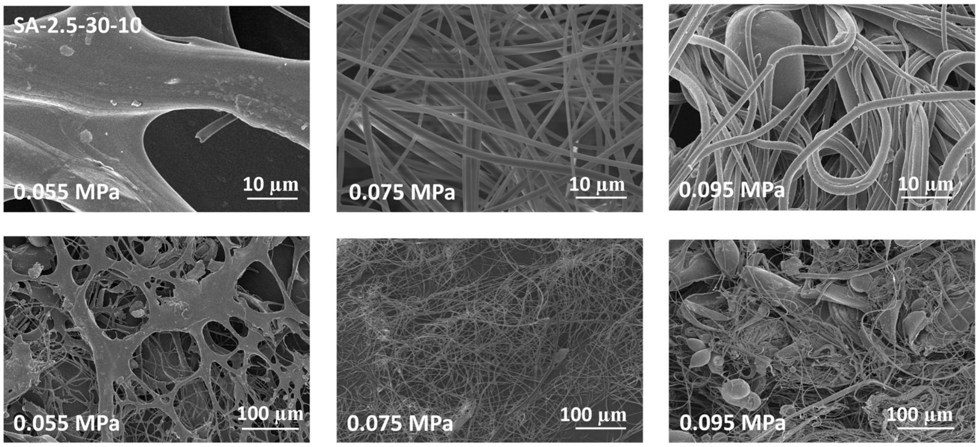

Figure S11. SEM images of blowing-spinning fibrous membranes (SA-2.5-30-10) under different air pressures.

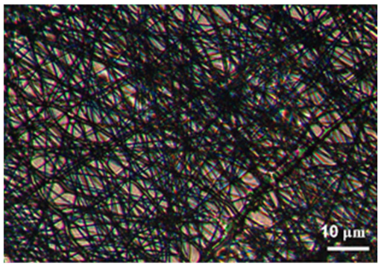

Figure S12. The microscopic image of SA-2.5-30-10 fibrous membrane.

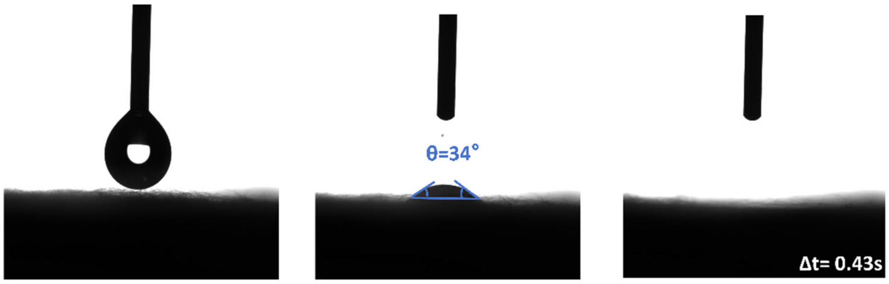

Figure S13. Photo of the surface wettability behavior for water of the cross-linked SA-2.5-30-10 fibrous membranes.

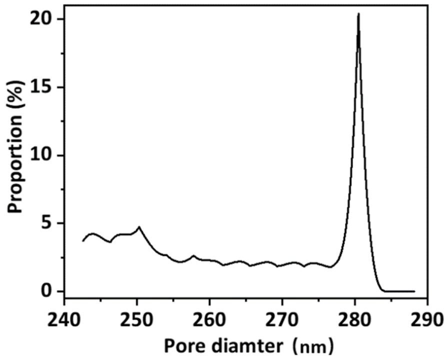

Figure S14. Pore size distribution of SA-2.5-30-10 fibrous membranes.

Table S1. Non-Newtonian index n_s of SA-c_p-f_{PEO}-f_{EtOH} solutions.

SA-c_p-f_{PEO}-f_{EtOH}	n_s
SA-2.5-30-0	0.29466
SA-2.5-30-5	0.24592
SA-2.5-30-10	0.21764
SA-2.5-30-15	0.23187
SA-2.5-30-20	0.22096
SA-1.5-30-10	0.39841
SA-2-30-10	0.28039
SA-3-30-10	0.20307
SA-2.5-10-10	0.26831
SA-2.5-20-10	0.22534

Table S2. Surface tension and initial filament diameter of SA-c_p-f_{PEO}-f_{EtOH} solutions.

SA-c_p-f_{PEO}-f_{EtOH}	σ (mN/m)	D_0 (mm)
SA-2.5-30-0	58.16	2.36766
SA-2.5-30-5	52.27	1.38825
SA-2.5-30-10	44.48	1.58441
SA-2.5-30-15	37.52	2.68335
SA-1.5-30-10	55.11	1.47203
SA-2-30-10	48.64	1.71786
SA-3-30-10	30.68	1.7034
SA-2.5-10-10	46.02	1.20234
SA-2.5-20-10	45.86	1.34977

Table S3. Fitting parameters of SA-c_p-f_{PEO}-f_{EtOH} solutions.

SA-c_p-f_{PEO}-f_{EtOH}	λ_E	η_E^∞	η_{sp}^∞	η_0	Tr^∞
SA-2.5-30-0	0.81	3137.43	1099117.279	33.6549	91.119
SA-2.5-30-5	1.236	5088.07	1531559.606	48.1936	104.874
SA-2.5-30-10	1.516	4758.23	1250635.815	74.5	67.485
SA-2.5-30-15	2.0442	-	-	93.3	-
SA-1.5-30-10	0.259	-	-	10.1	-
SA-2-30-10	0.279	-	-	27.4	-
SA-3-30-10	3.50	-	-	123.4	-
SA-2.5-10-10	0.212	-	-	52.5	-
SA-2.5-20-10	0.9703	-	-	61.4	-
